# RNA polymerase mapping in plants identifies intergenic regulatory elements enriched in causal variants

**DOI:** 10.1093/g3journal/jkab273

**Published:** 2021-09-06

**Authors:** Roberto Lozano, Gregory T Booth, Bilan Yonis Omar, Bo Li, Edward S Buckler, John T Lis, Dunia Pino del Carpio, Jean-Luc Jannink

**Affiliations:** 1 Plant Breeding and Genetics, School of Integrative Plant Science, Cornell University, Ithaca, NY 14853, USA; 2 Department of Molecular Biology and Genetics, Cornell University, Ithaca, NY 14853, USA; 3 Montpellier SupAgro, Montpellier Cedex 02 34060, France; 4 State Key Laboratory of Plant Genomics and National Center for Plant Gene Research, Institute of Genetics and Developmental Biology, Chinese Academy of Science, Beijing 100101, China; 5 Institute for Genomic Diversity, Cornell University, Ithaca, NY 14853, USA; 6 United States Department of Agriculture, Agricultural Research Service (USDA-ARS) R.W. Holley Center for Agriculture and Health, Ithaca, NY 14853, USA

**Keywords:** PRO-seq, GRO-seq, promoter, enhancer, regulatory elements, pausing

## Abstract

Control of gene expression is fundamental at every level of cell function. Promoter-proximal pausing and divergent transcription at promoters and enhancers, which are prominent features in animals, have only been studied in a handful of research experiments in plants. PRO-Seq analysis in cassava (*Manihot esculenta*) identified peaks of transcriptionally engaged RNA polymerase at both the 5′ and 3′ end of genes, consistent with paused or slowly moving Polymerase. In addition, we identified divergent transcription at intergenic sites. A full genome search for bi-directional transcription using an algorithm for enhancer detection developed in mammals (dREG) identified many intergenic regulatory element (IRE) candidates. These sites showed distinct patterns of methylation and nucleotide conservation based on genomic evolutionary rate profiling (GERP). SNPs within these IRE candidates explained significantly more variation in fitness and root composition than SNPs in chromosomal segments randomly ascertained from the same intergenic distribution, strongly suggesting a functional importance of these sites. Maize GRO-Seq data showed RNA polymerase occupancy at IREs consistent with patterns in cassava. Furthermore, these IREs in maize significantly overlapped with sites previously identified on the basis of open chromatin, histone marks, and methylation, and were enriched for reported eQTL. Our results suggest that bidirectional transcription can identify intergenic genomic regions in plants that play an important role in transcription regulation and whose identification has the potential to aid crop improvement.

## Introduction

Gene expression in plants is a highly regulated process controlling the production of coding and noncoding RNA molecules. Proper regulation of expression is central to the development and phenotypic plasticity. The dynamics of transcriptional regulation have been extensively studied in several model organisms, including humans, yeast, and fruit flies (Jennings 2013). These studies have revealed a complex network of molecular elements that orchestrate gene expression patterns and thereby shape the transcriptional landscape of each organism. Failure in gene regulation control can have detrimental effects on development and lead to disease or other disorders (Adelman and Lis 2012).

Nascent RNA sequencing techniques such as Global nuclear Run-On sequencing (GRO-seq) (Core *et al.* 2008) or Precision nuclear Run-On sequencing (PRO-seq) (Kwak *et al.* 2013; Mahat *et al.* 2016) have been used to map and quantify transcriptionally engaged polymerase density. These techniques have identified promoter-proximal pausing of Polymerase II (Pol II) and bi-directional transcription (Sigova *et al.* 2013) as widespread phenomena in metazoans (Rennie *et al.* 2018). The pausing of elongating Pol II occurs shortly after the Pre-Initiation Complex is assembled and initiation has occurred (Adelman and Lis 2012). Promoter-proximal pausing has been suggested as a mechanism to tune the expression of specific genes in response to external regulatory signals and might also play a role in stabilizing the open chromatin state around promoter regions (Adelman and Lis 2012).

Enhancers are key eukaryotic regulatory elements that control spatiotemporal gene expression and are especially important during development (Weber *et al.* 2016). Studies in mammals have shown that enhancers produce short unstable RNAs known as eRNAs (Kim *et al.* 2010, 2015). While the specific role of eRNAs is not clear, the presence of eRNAs supports a more unified model of transcription initiation between enhancers and promoters (Li *et al.* 2013; Core *et al.* 2014; Sigova *et al.* 2015).

Only a few studies on a limited number of species (Erhard *et al.* 2015; Hetzel *et al.* 2016; Zhu *et al.* 2018) have investigated whether bidirectional transcription at promoters and enhancers is present also in plants. GRO-seq analysis in Arabidopsis seedlings showed little support for this phenomenon (Hetzel *et al.* 2016). Similarly, Zhu *et al.* (2018) also concluded that transcription would be unidirectional in Arabidopsis. Prominent 3′ accumulation of RNA polymerase was observed in both maize and Arabidopsis. These data suggest that gene regulation in plants may have diverged from what is observed in other eukaryotes, reflecting a different evolutionary approach to gene regulation within the plant kingdom (Hetzel *et al.* 2016). A recent study, however, used Arabidopsis mutants defective in nuclear RNA decay to characterize Arabidopsis transcription (Thieffry *et al.* 2020). They found that divergent transcription at genes was uncommon but not absent. In addition, they found evidence of bidirectional transcription at intergenic regions that share many of the mammalian enhancer region signatures.

The objective of this study was to characterize nascent transcription in cassava (*Manihot esculenta*) and maize (*Zea mays*), and to reveal if promoter-proximal pausing and bidirectional transcription at intergenic regions are present in these species. To do so, we quantified nascent transcription in cassava and maize using PRO-seq and re-analyzed a maize GRO-seq dataset (Erhard *et al.* 2015). We showed that cassava’s nascent transcriptome contains features of transcriptional regulation that were not present or detected in previous plant experiments, including promoter-proximal pausing and bidirectional transcription at intergenic regions. We used the bidirectional transcription profiles at intergenic regions to identify intergenic regulatory elements (IREs) in both cassava and maize. Most importantly, in cassava, we demonstrated that these intergenic regions contributed disproportionately to the SNP heritability of several complex agronomic traits. Similarly, we found that the IRE candidates in maize were enriched in eQTLs and co-localized with previously identified enhancers.

## Materials and methods

### Plant materials and nuclei isolation

Cassava accession “Nase 3” (synonymous with “IITA-TMS-IBA30572” and “Migyera”) cuttings were grown in tubes containing enriched medium. Tubes were placed in growth chambers with 12 hours of light at 30°C for 6 weeks before tissue collection. Stem and leaves of approximately 25 gr were ground with liquid nitrogen to a fine powder using a mortar and pestle. The resulting powder was transferred to a coffee grinder containing cold 1X NIB buffer. We then used the CelLytica PN Plant nuclei isolation/extraction kit (Sigma-Aldrich) following the instructions for the “Highly pure preparation of Nuclei.” The resulting solutions were frozen in liquid N_2_ and stored at −80°C. Most of the nuclei extraction protocol took place in a cold room (4°C) with all reagents on ice. A fraction of the nuclei preparations were stained with DAPI (4′,6-diamidino-2-phenylindole) and visualized under a fluorescence microscope to test for concentration and nuclei integrity.

Maize inbred line B73 seeds were put into growth chambers. Shoots were collected 9 days after germination. Around 10 grams of plant tissue were ground with liquid nitrogen to a fine powder. Five grams of ground tissue were transferred into 50 ml fresh SEB extraction buffer (2.0% PVP, 10%TKE, 500 mM sucrose, 4 mM spermidine, 1 mM spermine, 2.5% β-mercaptoethanol), incubated on ice for 20 minutes, and then filtered through 2 layers of 100um nylon mesh. Triton X-100 was then added to a final concentration of 0.5% and incubated on ice for another 10 minutes. Then the lysate was centrifuged at 2000 rcf for 15 minutes at 4°C and the supernatant was recovered. The pellet was suspended in another 25 ml SEB extraction buffer and centrifuged again at 2000 rcf for 15 minutes. We added 10 ml nuclei storage buffer (50 mM Tris-Cl, 50% glycerol, 5 mM MgCl2, 0.1 mM EDTA, 0.5 mM DTT) in the pellet and centrifuged at 2000 rcf for 5 minutes at 4°C. This step was repeated using 1 ml nuclei storage buffer. Finally, the pellet was resuspended and stored in 105 µl nuclei storage buffer. The protocol was conducted in a cold room (4°C).

### Pro-seq library preparation and sequencing

The PRO-seq protocol was performed as described by Mahat *et al.* (2016). Briefly, nuclei isolation washed away endogenous nucleotides, halting elongating RNA polymerases bound to chromatin. Precision run-on reactions were performed in the presence of equimolar amounts of unaltered ATP and GTP, as well as biotin-11-CTP and biotin-11-UTP (Perkin-Elmer). Notably, this two-biotin run-on will produce polymerase profiles with slightly reduced (∼ 2–4 bp) resolution compared to a more typical four-biotin run-on, given that RNA polymerases will primarily stall when incorporating the modified CTP and UTP (Kwak *et al.* 2013). RNA was extracted and base-hydrolyzed with NaOH. Hydrolyzed, biotin-labeled nascent RNAs were passed through a RNase-free P-30 spin column (Bio-Rad) and then enriched using M-280 streptavidin Dynabeads (Invitrogen). T4 RNA ligase 1 (NEB) was used to attach a 3′ RNA adaptor containing a six-nucleotide unique molecular index (UMI) for the removal of duplicate sequences produced by PCR (5′-/5Phos/NNNNNNGAUCGUCGGACUGUAGAACUCUGAAC/Inverted dT/-3′). After a second biotin-enrichment, RNAs were submitted to RNA 5′ Pyrophosphohydrolase (RppH, NEB) treatment for 5′ de-capping, and then 5′ phosphorylation with T4 polynucleotide kinase (T4 PNK, NEB), before ligation of the 5′ RNA adaptor (5′-CCUUGGCACCCGAGAAUUCCA-3′). Reverse transcription was performed with SSIII RT (Invitrogen) after a third biotin-enrichment. The cDNAs produced were PCR amplified for 13 cycles with Phusion polymerase (NEB) and size selected (120–400 bp) before sequencing. This protocol generated strand-specific libraries with every read starting from the 3′ end of the RNA. The RNA adapters used are TruSeq-compatible and libraries were reverse transcribed and amplified using primers from the Illumina TruSeq small RNA sequencing kit. Amplified libraries were assessed for quality on a bioanalyzer prior to sequencing on a HiSeq2500 with 100 bp single reads.

### Analysis of NGS data

#### Processing and read alignment

The fastq files were scanned for any residual adapter sequence (5′-TGGAATTCTCGGGTGCCAAGG -3′) using fastx_clipper from the FASTX_toolkit (http://hannonlab.cshl.edu/fastx_toolkit/), and the 3′ molecular barcode was removed. Reads were trimmed to a maximum length of 36 bp, and the reverse complement was calculated because the HiSeq apparatus starts sequencing from the 5′ end. All downstream alignments were performed using Bowtie2 (Langmead and Salzberg 2012). Because the PRO-seq method is not exclusive to transcripts produced by the nuclear RNA Polymerase II, the reads were aligned to the chloroplast genome to eliminate organellar transcripts. The remaining reads were mapped to the *M.* *esculenta* reference genome v6.1, the maize genome AGPv4 or the Arabidopsis genome TAIR10 (www.phytozome.com). Low-quality alignments were filtered and only reads mapping once to the genome were considered for further analysis (Supplementary Table S1). Bedtools (Quinlan and Quinlan 2014) was used to get bedgraph files reporting only the number of 3′ end reads at each position. Finally, bigwig files were obtained from the bedgraph files using kentUtils (https://github.com/ENCODE-DCC/kentUtils).

#### Pro-seq read distribution

The percentage of the cassava genome transcribed was calculated using bedtools (Supplementary Figure S1A). A saturation curve, which calculates the number of unique positions covered as a function of read depth was obtained using the bed-metric scripts (https://github.com/corcra/bed-metric.git) (Supplementary Figure S1). Normalized BigWig files representing the mapped reads were used to visualize each strand of the genome separately in the Integrative Genomics Viewer (IGV) (Thorvaldsdottir *et al.* 2013). The Metagene plots, histograms, peak scanning, and gene expression values were generated using the HOMER software (Heinz *et al.* 2010) and meta-gene maker (https://github.com/bdo311/metagene-maker). Since the cassava genome is not readily available to work with HOMER, feature annotations were created separately, and the HOMER config files were modified. We approximated the transcription start site (TSS) as the beginning of the 5′ UTR because the cassava genome annotation lacked precise TSS annotations. The same approach was used for Arabidopsis and Maize genomes.

#### Quantifying pausing and divergent transcription

Genes were ranked based on their pausing index. The pausing indices were calculated as previously described (Chen *et al.* 2015; Williams *et al.* 2015). Pausing index is defined as the ratio of PolII signal density near a gene promoter to signal density within the gene body. Specifically, the average coverage in the promoter region (100 upstream of the TSS to 300 downstream of the TSS) divided by the average coverage of the gene body (300 bp downstream of the TSS to the Polyadenylation site, PAS). Divergent transcription indices were calculated similarly by taking the average coverage of the upstream promoter region (from 1 kb upstream of the TSS to the TSS) in the antisense strand (with respect to the gene) divided by the average coverage of the TSS proximal region (300 bp upstream the TSS to 300 bp downstream the TSS) in the sense strand. We modeled the degree of promoter-proximal pausing and divergent transcription as measured by these indices using different factors including gene length, gene expression measured in RPKM (Reads per Kb of genic region per million mapped reads), cDNA length and number of exons using a linear model.

#### GERP and methylation data

The GERP scores for conservation were calculated using different species from the Malpighiales clade, including rubber (*H. brasiliensis*), jatropha (*Jatropha curcas*), castor bean (*Ricinus communis*), willow (*Salix purpurea*), flax (*Linum usitatissimum*), and poplar (*Populus trichocarpa*) as previously described by Ramu *et al.* (2017). Whole-genome methylation data for cassava was available from Wang *et al.* (2015).

### Genomic partitioning in cassava

Genomic Partitioning is a method to explore the genetic architecture of complex traits (Yang *et al.* 2011; Speed *et al.* 2017). In this step, we calculated the heritability contribution from the IREs in the cassava genome and compared it with a random set of DNA regions of similar size and occupying a similar distribution across the cassava chromosomes.

#### Field-evaluated phenotypes and germplasm

We analyzed data from the IITA cassava breeding program in Nigeria, including a fraction (689 clones, *i.e.*, genetically unique individuals, each of which is clonally propagated) of the Genetic Gain (GG) collection, which comprises 709 elite and historically important clones. Along with these, we analyzed 2302 clones developed as the cycle 1 of IITA’s Genomic Selection (GS) breeding program. In total, 3011 clones were used as the source for phenotypes. For further details on the populations used, see Wolfe *et al.* (2016b, 2017). We analyzed 5 traits: Dry matter content (DM), mean cassava mosaic disease severity (MCMDS), root number (RTNO), shoot weight (STWT), and fresh yield (FYLD). The phenotyping trials used in this study have been described before and all phenotype data is provided in Supplementary Table S2.

#### Genotype data

Genotyping-by-sequencing (GBS) libraries were prepared as described previously (Elshire *et al.* 2011). Marker genotypes were called using the TASSEL-GBS discovery pipeline (Glaubitz *et al.* 2014) using the *M.* *esculenta* genome assembly v6.1 (www.phytozome.net). The GBS markers were combined with the Cassava HapMap v2.0 variants from 241 deep-sequenced cassava accessions (Ramu *et al.* 2017) to impute variants on all clones to whole-genome sequence level in a single step with IMPUTE2 (Howie *et al.* 2009, 2011). The imputation procedure was performed as in Lozano *et al.* (2017) where the number of haplotypes used as the reference panel was set to 400, the effective population size (Ne) to 1000, and the imputation window to 5 Mb. The resulting Oxford files were converted into the Plink (Chang *et al.* 2015) binary format. In total, 3 million variants with a quality info score higher than 0.3 and Minor Allele Frequency (MAF) >0.01 were obtained for the 3011 individuals used in this study.

#### Variance component estimation

Genomic partitioning analyses are imprecise in highly related populations because of high LD between partitions. We mitigated this problem by eliminating markers from the rest of the genome (ROG) in high LD with the 9665 cassava IREs (markers were removed that had allelic *r*^2^ > 0.9 and were closer than 100 kb to IRE markers). We also built 10 random sets of 9665 regions with the same average length and approximate distribution in the genome as these elements. As with the IREs, random sets were forced to be outside any annotated gene (Supplementary Figure S2), and markers from the ROG in close physical distance and high LD were removed.

Genomic relationship matrices (GRMs) were calculated for focal (*i.e.*, either IREs or random sets) and ROG genomic partitions using the software LDAK5 following the ideas of Speed *et al.* (2017). Briefly, LDAK5 relationship matrices control short-range LD by assigning marker weights. Markers residing in low LD regions will have higher weights and are assumed to contribute more than markers in high LD regions. The LDAK5 model also assumes that a SNP’s heritability depends on its MAF, using an α value set to −0.25 as suggested in Speed *et al.* (2017). Finally, LDAK5 considers genotyping uncertainty as higher-quality observed markers that should contribute more than poorly imputed markers. GRMs were calculated for the IREs partition and the ROG partition. Separate analyses used GRMs based on the ten random sets. Python scripts for these analyses can be accessed at the GitHub repository associated with this article.

The model fit to calculate the variance components was specified in matrix notation as:

$$Y\;=\;X\beta \;+Z_{\mathrm{loc}.yr}l\;+\;Zg\;+\;Zh\;+\;e$$

where ***Y*** represents a vector with raw phenotypic observations, ***β*** represents the intercept and **X** is a vector of ones. The random effects include a single intercept for each location-year combination in which phenotypic trials took place and where ***l ∼ N(0, Iσ^2^_l_)*** where ***I*** is the identity matrix and ***σ^2^_l_*** is the associated variance component. The genetic variance components include ***g*** and ***h*** where ***g ∼ N(0, GRM_F_σ^2^_g_)***and ***h ∼ N(0, GRM_R_σ^2^_h_)***. These two terms have a known covariance structure calculated using LDAK5 for the focal (***GRM_F_***) or ROG (***GRM_R_***) partitions. The incidence matrices **Z_*loc*__.__*yr*_**, and **Z** relate observations to the levels of trials and clones, respectively. Variance components were estimated using the “emmremlMultikernel” function implemented in the “EMMREML” R package (Akdemir and Okeke 2015).

### Colocalization of eQTLs with regulatory element candidates identified by GRO/PRO-seq/dREG in maize

We used a list of 61k eQTLs found in maize kernels. The identification of the eQTLs was performed using the B73 maize reference genome AGPv2. We then uplifted the position of the eQTLs to AGPv3 and then AGPv4 using Crossmap (Zhao *et al.* 2014). We removed all the eQTLs positioned within 3k of any gene. Using this criterion we ended up with 7271 intergenic eQTLs. This dataset was compared with the enhancer candidate regions identified by dREG using bedtools intersect (Quinlan and Quinlan 2014) where we consider as a match any overlap with 50% or more of the enhancer sequence. We also generated 10 k random sets of intergenic regions with the same size distribution as the enhancer candidates identified by dREG. Using this set we calculated the empirical distribution of regions matching to eQTLs just by chance.

## Results

To investigate nascent RNA profiles in plants, we generated PRO-seq libraries in cassava and maize seedlings. To complement our study we re-analyzed previously published GRO-seq datasets available in maize and Arabidopsis (Supplementary Table S1). For clarity, the four main libraries used through this study will be referred to as PRO-cassava, PRO-maize, GRO-maize (Erhard *et al.* 2015), and GRO-arabidopsis (Hetzel *et al.* 2016).

### Polymerase accumulation around coding segments differs between plant species

**Figure 1:**
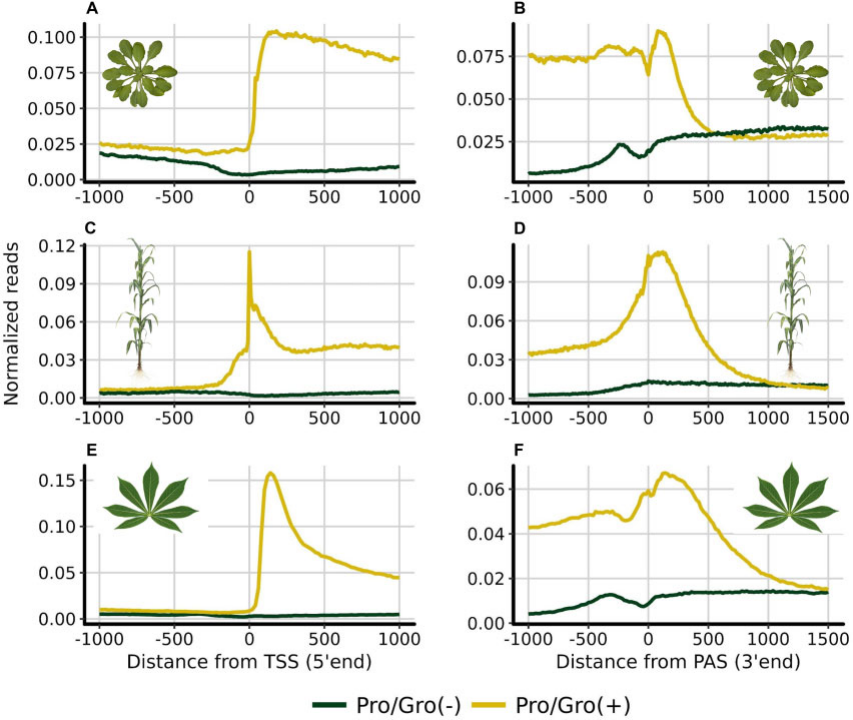
Accumulation of Pro-seq reads around the Transcription Start Site and Polyadenylation sites of three different plant species. Metaplot of GRO/PRO-seq signal from annotated genes normalized for reads per bp per gene in *Arabidopsis thaliana* (GRO-seq, *n* = 28,775) (A, B), *Zea mays* (PRO-seq, *n* = 38,943) (C, D) and *M. esculenta* (PRO-seq, *n* = 31,895). Reads were aligned to the TSS and the PAS in both sense (yellow) and antisense (green) directions relative to the direction of gene transcription (E, F). Prominent promoter-proximal pausing is shown in *M. esculenta*, and to some degree in maize, but it is not present at all in Arabidopsis as previously reported (Hetzel *et al.* 2016). Accumulation of RNA polymerase at the 3′ end of the genes is a common feature in the three plant species.

We explored the accumulation of engaged RNA polymerase around the gene bodies of maize, cassava, and Arabidopsis by mapping reads generated by GRO/PRO-seq to the reference genome of each species. In agreement with Hetzel *et al.* (2016), GRO-arabidopsis lacked 5′ pausing (Figure 1A) and, instead, showed accumulation of engaged polymerases at the 3′ end of each gene (Figure 1B). Analysis of PRO-maize showed 3′ pausing (Figure 1D) and a small accumulation of reads at the TSS (Figure 1C). That accumulation was consistent with GRO-maize, and thus generalized between the two techniques (Supplementary Figure S3). In contrast, PRO-cassava showed a clear pattern of both 5′ (Figure 1E) and 3′ pausing (Figure 1F). Out of the 24,532 genes that were expressed in cassava, 16,605 had a Pausing Index (PI) higher than two (Supplementary Figure S4, see Materials and Methods). While all three plant species demonstrated polymerase accumulation at the 3′ end of genes, each displayed a unique accumulation pattern in the promoter-proximal region. Unlike mammals, bi-directional transcription is uncommon among plant promoters (Figure 1), though several cassava genes exhibiting this behavior were identified (Supplementary Figure S5).

### Polymerase mapping in noncoding regions identifies intergenic regulatory elements (IRE) candidates in cassava

**Figure 2:**
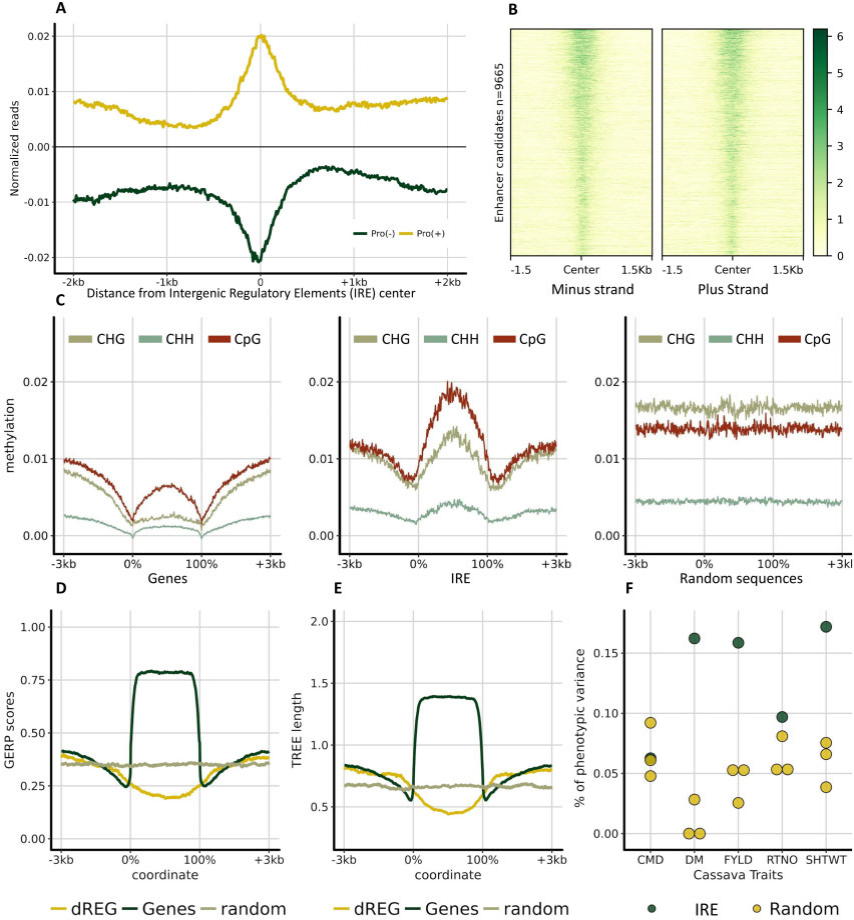
IREs in cassava have a particular methylation pattern, are evolutionary less conserved and explain more phenotypic variance than expected for several agronomic traits. (A) Pro-seq reads mapping around cassava IREs. Reads were sorted by strand and the normalized reads were plotted around the center of each candidate. (B) Heatmap representation of reads mapping to the enhancer candidate regions. The regions are sorted based on dREG scores. (C) Cassava methylation patterns for the three methylation contexts (CG, CHH, and CHG) were plotted around the genic regions, IREs, and a set of random sequences. The random set has the same number and length distribution as the IREs. Genomic regions were scaled (0–100%) for visualization. (D) GERP scores and corresponding tree lengths (E) were also plotted around the IREs (dREG), Genes, and random set of regions. (F) Genomic Partitioning of complex agronomic traits (DM: Dry matter content; FYLD: Fresh yield; RTNO: Root number; SHTWT: Shoot weight) and a disease trait (CMD: Severity of Cassava Mosaic Disease). Relationship matrices were calculated using SNP markers within the enhancer candidate regions using the LDAK5 model and variance components were estimated using EMMREML.

We mapped PRO-seq peaks outside coding regions (more than 3 kb from the 5′ UTR or 3′ UTR of any gene) in the cassava genome. We identified ∼2000 peaks in intergenic regions using Homer’s (Heinz *et al.* 2010) Chip-seq peak finder. The regions identified showed a clear bi-directional transcription, similar to that observed in mammalian and other metazoan enhancers (Supplementary Figure S6). Given the resemblance of these elements to mammalian enhancers, we used discriminative regulatory-element detection (dREG) (Danko *et al.* 2015), a support vector regression algorithm trained to detect enhancers and promoters from GRO-seq mapped reads. We annotated 34,000 of these regions across the cassava genome of which 16,800 were located in intergenic regions, and 9665 were at least 1 kb away from any gene. This set of 9665 regions, which we refer to as IREs (Supplementary File S1), showed a clear bidirectional pattern of transcription (Figure 2, A and B, Supplementary Figure S7). We believe that these regions are enriched with enhancers and other regulatory elements.

Three independent lines of evidence supported the biological activity of these IRE regions. First, using DNA methylation data previously generated in the cassava cultivar TME7 (Wang *et al.* 2015), we observed profiles in the three DNA contexts, CG, CHG, and CHH, around the 9665 cassava IRE regions distinct from genic and random regions across the genome (Figure 2C). Second, genomic evolutionary rate profiling (GERP) (Cooper *et al.* 2005; Davydov *et al.* 2010; Ramu *et al.* 2017) of the IRE showed lower conservation of these regions than of random sequences across the genome, whereas coding regions are conserved (Figure 2, D and E). This low conservation agrees with observed patterns in mammals (Villar *et al.* 2015) where enhancers, unlike promoters, are rarely conserved and evolve rapidly.

Finally, to test the functional relevance of the plant IREs, we estimated the percentage of the SNP heritability (Speed *et al.* 2017) attributable to these regulatory element candidates as compared to randomly ascertained genomic regions with the same intergenic distribution. We set up genomic partitions (Yang *et al.* 2011; Gusev *et al.* 2014) separating the focal partition from the ROG partition, where the focal partition was either the IRE candidates or the random regions. We used 3011 cassava clones (*i.e.*, genetically unique individuals, but each clonally propagated for replicated evaluation) of the NextGen Cassava Breeding Project (Wolfe *et al.* 2017) evaluated for four quantitative agronomic traits: dry matter content (DM), fresh yield (FYLD), number of roots (RTNO), and shoot weight (SHTWT), and one disease trait whose genetic architecture is controlled primarily by a single resistance locus (Wolfe *et al.* 2016a): MCMDS. GRM for each partition was calculated using LDAK5, following recommendations made by Speed *et al.* (2017). For each partition, we fit a model estimating variances of effects distributed according to ROG and focal GRMs. The percentage of phenotypic variance explained by markers inside IRE regions was higher than random regions for all quantitative agronomic traits but not for the disease resistance trait (Figure 2F). There is no standard significance test for contrasting the alternative enhancer candidate hypothesis to the null model random set hypothesis (Deniz Akdemir, pers. comm.). Repeated sampling of the null model, however, shows nonoverlap of its distribution of variance component estimates with the point estimate of the alternative for all four quantitative agronomic traits. We only sampled the null model ten times, creating ten random kernels because the two-GRM one-step model was computationally intensive. Assuming independence among the four agronomic traits, the probability that all null models across all traits would explain less variance than the alternative, under the null hypothesis that random sets explain the same variance as IRE, would be (1/11)^4 = 6.83e-5. In fact, root number and fresh root yield are strongly correlated [0.65 to 0.80, (Okeke *et al.* 2017)] but are both uncorrelated to dry matter content and shoot weight. Thus, a conservative *P*-value for the hypothesis that IRE explains more variance in quantitative agronomic traits than random sets would be (1/11)^3 = 7.51e-4.

### IRE in maize co-localize with previously reported enhancer candidates

**Figure 3:**
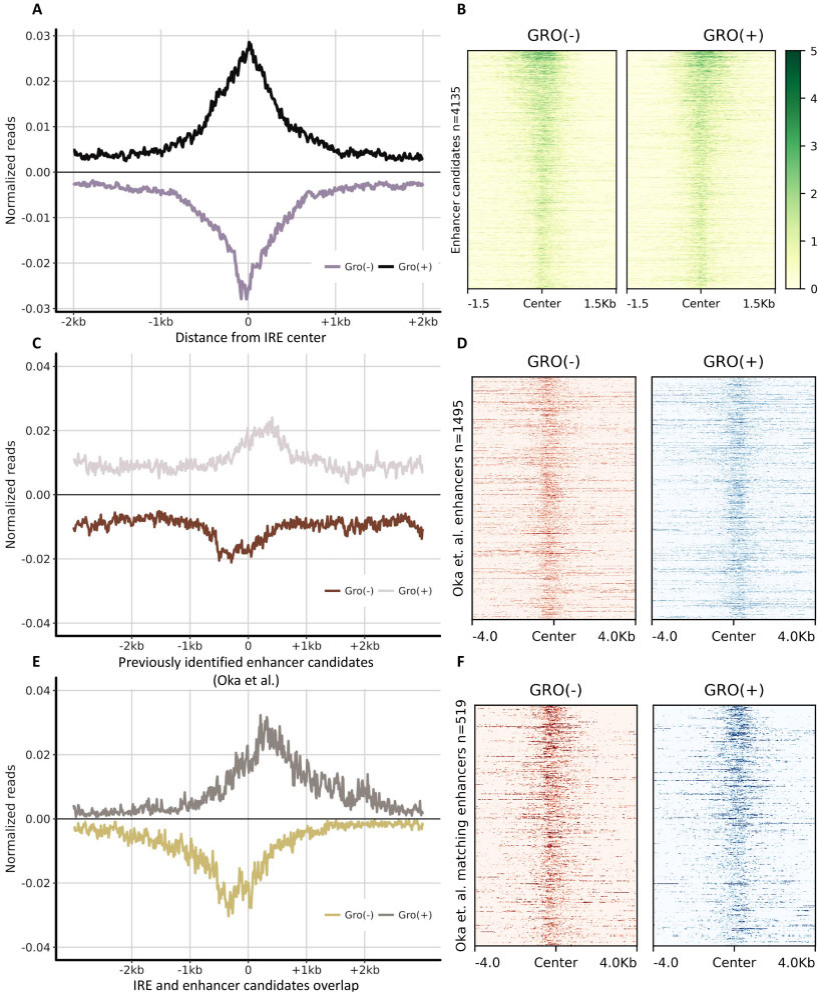
Enhancer candidates previously identified in Maize produce eRNAs. (A) GRO-seq reads (Erhard *et al.* 2015) mapping around maize IRE IREs detected by dREG. Reads were sorted by strand and the normalized reads were plotted around the center of each candidate (*n* = 4135). (B) Heatmap representation of reads mapping to the IREs. The regions are sorted based on dREG scores. (C) GRO-seq reads were mapped to the regions previously identified as enhancer candidates by Oka *et al.* (2017) based on methylation, histone marks, and chromatin accessibility (*n* = 1495). (E) A portion of the enhancer candidates (*n* = 519) reported by Oka *et al.* were also identified by dREG using just the GRO-seq reads. Heatmaps showing the GRO-seq signal of the regions in (C) and (E) are shown in (D) and (F), respectively.

In an attempt to extend our observations to other plants, we analyzed existing GRO-maize data similarly to our PRO-cassava data. We identified 4135 (Supplementary File S2) IREs using dREG and again observed a clear pattern of bi-directional transcription (Figure 3, A, and B). Our PRO-maize libraries were not used to identify enhancer candidates due to their lower number of mapped reads, an issue that affects the overall detection power of dREG (Supplementary Table S1). When enhancer candidates were identified using GRO-maize, however, PRO-maize reads aligned to those candidates also showed strong signal (Supplementary Figure S8).

It is unknown whether dREG can accurately predict plant enhancers. The traditional way to identify enhancer candidates is based on open chromatin sites as assessed by DNase-seq or ATAC-seq, together with histone modification marks. To test if dREG was able to tag previously reported enhancer candidates in maize, we analyzed the transcription pattern of 1495 (Supplementary File S3) enhancer candidates identified in a separate maize study (Oka *et al.* 2017) using an approach that integrated genome-wide methylation data, chromatin accessibility (DNase-seq), and histone marks (H3K9ac). Metaplots of the GRO-maize reads mapped against these candidates also showed bi-directional transcription (Figure 3, C and E). Moreover, 519 (Supplementary File S4) of the 1,495 IRE (∼35%) showed significant levels of bi-directional transcription and were independently identified by dREG (Figure 3, D and F).

To further characterize the IRE identified by dREG and explore whether they house more phenotypically relevant variants, we compared our results to a list of previously identified expression Quantitative Trait Loci (eQTLs) in maize kernels (Liu *et al.* 2017). We expected that IRE would be enriched with intergenic eQTLs, as enhancers regulate messenger RNA expression levels. We found that 372 out of the 4135 intergenic enhancer candidates (8.9%) identified in our study matched with eQTLs reported by Liu *et al.* (2017). To create a null distribution of the overlap, we calculated the empirical distribution of eQTL matches against 10,000 random sets of 4135 random intergenic regions with the same size as our IREs (Supplementary Figure S9). The observed overlap (8.9%) was far outside the range of this null distribution (mean <1%).

We also compared the IRE regions in maize to the DNase-seq-peaks found by Oka *et al.* (2017) in both husk and V2 inner stem (V2-IST) tissues. The husk and V2-IST have 4196 and 4529 intergenic open chromatin sites (3 kb away from any gene) and they share 3206 common sites. We found 682 and 750 common regions in husk and V2-IST, respectively, relative to our 4135 IREs (Supplementary Figure S10). We expect that the intersection would be larger if the DNase-seq had been performed in the same tissue, genetic background, and developmental stage as the GRO-maize. As previously demonstrated, the open chromatin space in maize varies greatly among different tissues (Rodgers-Melnick *et al.* 2016). Finally, we compared the levels of eQTL enrichment among the categories defined by the intersection of the open chromatin data and our IRE candidates. For both husk and V2-IST tissues, a marginally larger percentage of eQTL-IRE colocalization occurred when the IREs identified with dREG were located within open-chromatin regions (Supplementary Figure S10). This, however, has the caveat that tissues were not matched between experiments.

## Discussion

The nascent transcriptome of cassava, as revealed by PRO-seq, showed features of transcriptional regulation that were not present or detected in previous plant experiments, including promoter-proximal pausing and the presence of bidirectional transcription at IRE.

### Promoter-proximal pausing

We note that plants, like yeast, lack the Negative ELongation Factor (NELF), which is likely required for a kinase-regulated release of paused Pol II (Narita *et al.* 2003). Thus, this enrichment of Pol II around the TSS may reflect a related maturation checkpoint observed in fission yeast (Booth *et al.* 2018).

We found that the patterns of transcripts around the TSSs were strikingly different in Arabidopsis, maize, and cassava. In rice, Joly-Lopez *et al.* (2020) mapped PRO-seq reads around protein-coding genes and, similar to cassava, found a clear pattern of promoter pausing and accumulation of transcription at the 3′ end of the genes. While these differences might be due to issues in their genome annotations, independent studies in Arabidopsis have shown contradictory results when analyzing GRO-seq data, some studies supporting its presence (Liu *et al.* 2018; Zhu *et al.* 2018) at least in a portion of the genes while others did not find evidence of it (Hetzel *et al.* 2016). We think that these discrepancies have to be reconciled with more replicates in different plant species, tissues, and conditions.

### Intergenic regulatory elements (IRE)

IRE regions were shown in cassava to have low levels of evolutionary conservation, a bi-directional pattern of transcription, and a specific DNA methylation profile. It is worth mentioning that PRO-seq cannot differentiate between Pol II and other polymerases (*i.e.*, Pol IV/V). Some of the transcriptional activity observed in the IREs might be driven by RNA polymerase dependent DNA methylation (RdDM). Thus, we expect the IRE regions to not only capture enhancers but also regulatory RNAs (mirRNAs, lnRNAs, and iRNAs) and transposons. This would partially explain the high methylation profiles and low evolutionary conservation at the IRE sites.

Most importantly, we showed that the IRE identified in the cassava genome contributed disproportionately to fitness and root composition variation. The only trait evaluated that did not show this behavior was a disease trait. This was expected as plant disease resistance is often conditioned by genes that cause recognition of infection rather than by differential expression (Jones and Dangl 2006). In contrast, fitness-related quantitative traits can be strongly affected by gene regulation (Kremling *et al.* 2018). These results suggest that the IRE identified in the cassava genome causally affect plant phenotypes by modulating gene transcription. Thus, the identification of these regions shows an important new way forward in prioritizing genomic regions for use in crop improvement.

In mammals, there are various enhancer states enriched with different histone marks: poised or inactive enhancers, primed enhancers, and active enhancers (Meng and Bartholomew 2018). Previous evidence suggests transcription at enhancer sites might even be a signature of an enhancer active state (Kim *et al.* 2010; Andersson *et al.* 2014). While we believe we have unique and valuable data to contribute to this discussion we cannot at this stage definitively answer the question of whether one type of identification (open chromatin *vs* GRO/PRO-seq dREG) leads to a superset or subset of sites relative to the other. We clearly show, however, that bi-directionally transcribed regions in maize have the characteristic of active enhancers of affecting gene transcription.

Recently, Ricci *et al.* (2019) did an extensive annotation of long-range trancriptional cis-regulatory elements (CREs) in the maize genome using ATAC-seq and histone marks. They identified that 1% of the maize genome were Accessible Chromatin Regions (ACRs), and 32% of those were >2 kb away from the nearest gene. We found that 31% of the regions identified in our study using dREG/PRO-seq co-located with distal ACRs identified in the aforementioned research. The same research also tried to capture specific CRE-gene loops using Hi-C, and we found that 17% of the distal edges of the loops identified also co-located with the IRE found here.

The ability of PRO-seq to detect functional intergenic regions has also been explored in rice. While building a fitness consequence map of the rice genome Joly-Lopez *et al.* (2020) observed that putative enhancers identified using PRO-seq/dREG (similar to this study) shared “enhancer-type characteristics” including enrichment for open chromatin sites, asymmetrically co-located H3K27ac marks and enrichment for DNA motifs found in open chromatin sites. We believe that the results in cassava, maize, and rice are evidence that, even though the IREs identified using dREG/PRO-seq might be capturing signals from other polymerases, IREs are enriched for real CREs.

Transcription of genomic enhancers was first described in 1992 (Tuan *et al.* 1992), but the lack of adequate technology prevented further research on the subject until the late 2000’s (Kim and Shiekhattar 2015; Long *et al.* 2016). While direct functions have been proposed for eRNAs as regulators of gene expression in metazoans (Chen *et al.* 2015; Kim and Shiekhattar 2015) there has been no evidence of this in plants to date. Previous work in Arabidopsis did not identify eRNAs (Hetzel *et al.* 2016), leading the authors to state that “if plants have enhancer elements, they rarely, if at all, produce transcripts.” Based on supporting research (Simpson *et al.* 1985; Shannon *et al.* 1991; Zhu *et al.* 2015; Weber *et al.* 2016), however, we believe the existence of plant enhancers is likely commonplace and independent of whether or not they are transcribed. Zhu *et al.* (2015) provided supporting evidence for this statement when they tested a small portion of nearly 10,000 enhancer candidates in *A. thaliana*: they validated 10 of the 14 enhancer candidates tested using a reporter assay. None of the Arabidopsis GRO-seq data, however, displayed strong evidence of transcription in the regions identified by Zhu *et al.* (2015) (Supplementary Figure S11).

The results reported herein cassava and maize suggest that plant transcriptional regulation may be more similar to that of mammals and other metazoans than previously thought. The lack of transcription previously observed in putative Arabidopsis enhancers may have been the result of different growth conditions, tissues, or even low read depth. Recent research, however, has shown that bidirectional transcription could be observed in 113 noncoding regions in Arabidopsis when using exosome mutants (Thieffry *et al.* 2020).

Furthermore, the genome size of Arabidopsis is very small. Maize, cassava, and rice have much greater noncoding space, allowing for the identification of IRE whose expression is not confounded by that of nearby genes. The identification of intergenic transcribed enhancers in cassava, maize, and rice but not in Arabidopsis is consistent with the functional space hypothesis proposed by Mei *et al.* (2018) that predicts more functional genomic segments (*e.g.*, enhancers and other regulatory elements) outside of genes in species with larger genomes. This hypothesis has recently been supported in a study that analyzed CRES in 13 plant species, where distal CRES were most abundant in larger and more complex genomes (Lu *et al.* 2019).

## Author contributions

R.L., D.P., B.Y., B.L., and G.B. performed experiments. R.L., D.P., J-L.J., J.L., and G.B. designed the experiment. E.B. provided intellectual input. R.L., G.B., J-L.J, J.L., and D.P. contributed to writing the manuscript. All the authors reviewed the final manuscript.

## Data availability

The raw sequencing files have been submitted to the NCBI Gene Expression Omnibus (GEO) under accession GSE114758. All other data needed to evaluate the conclusions in the paper are present in the paper or the supplementary materials. Custom scripts for the NGS analysis, metagene plots, and genomic partitioning, among others, have been made publicly available through the GitHub repository: https://github.com/tc-mustang/Pro-seq-Cassava. Supplementary Figures and Tables files are available at figshare: https://doi.org/10.25387/g3.14531340.
